# Optimized Identification of High-Grade Prostate Cancer by Combining Different PSA Molecular Forms and PSA Density in a Deep Learning Model

**DOI:** 10.3390/diagnostics11020335

**Published:** 2021-02-18

**Authors:** Francesco Gentile, Matteo Ferro, Bartolomeo Della Ventura, Evelina La Civita, Antonietta Liotti, Michele Cennamo, Dario Bruzzese, Raffaele Velotta, Daniela Terracciano

**Affiliations:** 1 Department of Experimental and Clinical Medicine, University Magna Graecia of Catanzaro, 88100 Catanzaro, Italy; 2 ElicaDea, Spinoff of Federico II University, 80131 Naples, Italy; matteo.ferro@ieo.it (M.F.); bartolomeo.dellaventura@unina.it (B.D.V.); e.lacivita@studenti.unina.it (E.L.C.); antonietta.liotti@unina.it (A.L.); dario.bruzzese@unina.it (D.B.); rvelotta@unina.it (R.V.); 3 Division of Urology, European Institute of Oncology (IEO), IRCCS, Via Ripamonti 435, 20141 Milan, Italy; 4 Department of Physics “Ettore Pancini”, University of Naples “Federico II”, Via Cintia 26 Ed. G, 80126 Naples, Italy; 5 Department of Translational Medical Sciences, University of Naples “Federico II”, 80131 Naples, Italy; michele.cennamo2@unina.it; 6 Department of Public Health, University of Naples “Federico II”, 80131 Naples, Italy

**Keywords:** prostate cancer, artificial neural network, PSA molecular forms, PSA density, tumor markers

## Abstract

After skin cancer, prostate cancer (PC) is the most common cancer among men. The gold standard for PC diagnosis is based on the PSA (prostate-specific antigen) test. Based on this preliminary screening, the physician decides whether to proceed with further tests, typically prostate biopsy, to confirm cancer and evaluate its aggressiveness. Nevertheless, the specificity of the PSA test is suboptimal and, as a result, about 75% of men who undergo a prostate biopsy do not have cancer even if they have elevated PSA levels. Overdiagnosis leads to unnecessary overtreatment of prostate cancer with undesirable side effects, such as incontinence, erectile dysfunction, infections, and pain. Here, we used artificial neuronal networks to develop models that can diagnose PC efficiently. The model receives as an input a panel of 4 clinical variables (total PSA, free PSA, p2PSA, and PSA density) plus age. The output of the model is an estimate of the Gleason score of the patient. After training on a dataset of 190 samples and optimization of the variables, the model achieved values of sensitivity as high as 86% and 89% specificity. The efficiency of the method can be improved even further by training the model on larger datasets.

## 1. Introduction

After skin cancer, prostate cancer (PC) is the most common among men [1,2]. The gold standard for the diagnosis of PC is based on the PSA (prostate-specific antigen) test [3,4]. Since PSA is produced by normal and malignant cells of the prostate gland, it is an organ-specific but not a cancer-specific biomarker. Based on this preliminary health screening, the physician decides on whether to proceed with further tests, typically prostate biopsy, to confirm or reject the initial hypothesis of a tumor, evaluate its aggressiveness, and assess its susceptibility to develop into a malignant cancer. Any elevation of the PSA above the cut-off is a cause for concern [3,4]. Nevertheless, various factors can cause a man’s PSA level to fluctuate, and a PSA test may either yield false-positive results or overdiagnosis of prostate cancer. Misinterpretation of diagnostic tests can expose patients to unnecessary treatments, including surgery and radiation therapy, with undesirable side effects, such as incontinence, erectile dysfunction, infections, and pain. Only about 25% of men who have a biopsy due to an elevated PSA level truly have cancer. The guidelines of the European Association of Urology (EAU-ESTRO-SIOG) for PC diagnosis recommend measuring PSA concentration and conduct a digital rectal examination (DRE) [5].

However, DRE has low sensitivity and PSA has low specificity, so the final diagnosis depends on biopsy. Consequently, a high rate of overdiagnosis and potentially overtreatment is observed and there is a strong clinical need of laboratory tests able to provide useful information to choose the best treatment option for each patient [6,7]. The increasing age of the worldwide population highlights this challenge as relevant, particularly in younger men who are at highest risk of the fearsome side effects of overtreatment. Therefore, it is mandatory to find diagnostic tools able to avoid unnecessary biopsy without missing clinically relevant PC.

We developed a deep learning algorithm that can identify aggressive forms of PC from a panel of 4 clinical variables plus the patient age. The levels of (i) total PSA (tPSA), (ii) free PSA (fPSA), (iii) the isoform-2 of proPSA (p2PSA), and (iv) PSA density were determined in 222 patients using automated immunometric assays described in the Methods of this article. The hypothesis that a similar set of variables plus age may represent most of the information on the state of PC is based on previous clinical studies and reports [8,9,10,11,12,13,14,15,16]. Thus, a model containing those variables can potentially diagnose PC with maximum achievable accuracy. Histological examination of samples has been used to validate the predictions of the algorithms.

The choice of using artificial neuronal networks (ANNs) to interpret data is motivated by the complexity of the problem. The number of potential variations that can result from a combination of 5 variables extracted by several different patients is very large. Describing such extremely complex data sets with deterministic models may be infeasible. A deterministic model may fail to keep up with the challenges that complex biological systems pose [17]. Contrary to deterministic models, artificial neural networks can discover the laws that determine the relationship among variables by analyzing the output of the system [18]. ANNs are statistical models partially modeled on biological neural networks. They are capable of modeling and processing nonlinear relationships between inputs and outputs in parallel. Architecturally, ANNs are modeled using layers of artificial neurons, or computational units able to receive input and apply an activation function along with a threshold to determine if messages are passed along, and these ultimately determine the output. In a simple model, the first layer is the input layer, followed by one hidden layer, and lastly by an output layer. Each layer can contain one or more neurons. Deep neuronal networks have more hidden layers, more neurons per layer, and more paths between neurons, endowing the networks with increased abstraction and problem-solving capabilities.

The proposed model could expedite the diagnostic pathway of PC patients by triaging high-grade cancers. Such a model predicts a biopsy Gleason score, which is the most powerful prognostic predictor for PC patients.

## 2. Methods

### 2.1. Study Population

**Figure 1:**
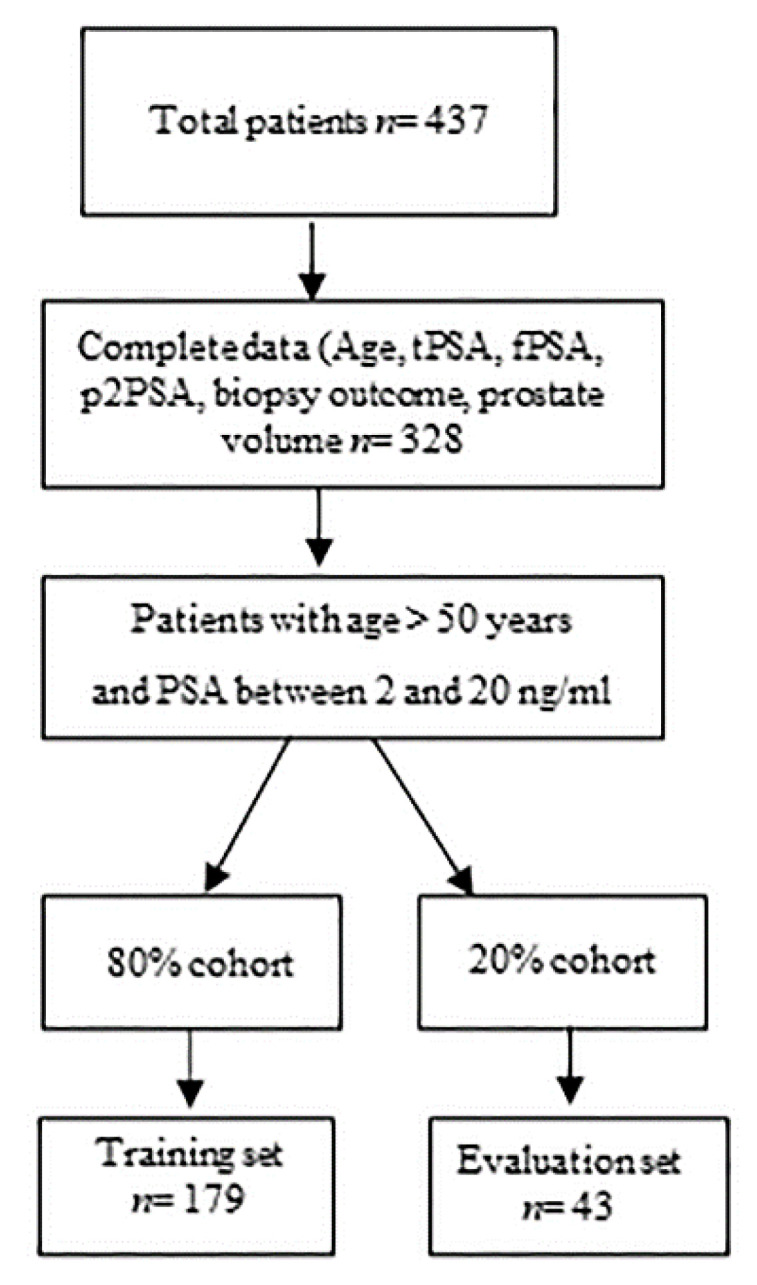
Summary of included patients.

Before prostate biopsy (minimum 16 cores), 437 subjects were enrolled in a prospective observational study, approved by the hospital ethics committee. Blood specimens were collected according to predetermined standard operating procedure [19]. Participants provided written approved consent. Ethical approval for this study was given by the institutional Ethics Committee of the University of Naples Federico II (118/20). Among these, 222 met eligibility criteria for this study: age over 50 years, no prior prostate surgery and biopsy, no bacterial acute or chronic prostatitis, no use of 5-α reductase inhibitors, PSA values between 2 and 20 ng/mL availability of serum samples and corresponding clinical data, and completion of at least a 16-core template biopsy after enrollment. The training cohort included 179 PC patients and the evaluation cohort 43. Figure 1 shows the summary of included patients. Table 1 shows descriptive characteristics of the study population.

### 2.2. Biomarker Measurement

Participants had blood drawn before DRE at each visit. Whole blood was allowed to clot before serum was separated by centrifugation. Serum aliquots were stored at −80 °C until samples were processed, according to Semjonow et al. [20]. Specimens were analyzed in a blinded fashion for PSA, fPSA, and p2PSA using a Access2 Immunoassay System analyzer (Beckman Coulter, Brea, CA, USA) calibrated against the WHO standard for PSA and fPSA. The analytical performance of the measurements assessed with control materials (Beckman Coulter) showed values within the allowed recommended limits. PSA density was calculated as PSA/prostate volume. Prostate volume was measured by transrectal ultrasound (TRUS). Patients underwent prostate biopsies according to a standardized institutional saturation scheme, which consisted of at least 16 needle biopsy cores obtained under transrectal ultrasound (TRUS) guidance. Primary and secondary Gleason scores were assigned by a single genitourinary pathologist blinded to the biomarker values, according to the 2005 consensus conference of the International Society of Urological Pathology definitions [21].

### 2.3. The Deep Learning Model

**Figure 2:**
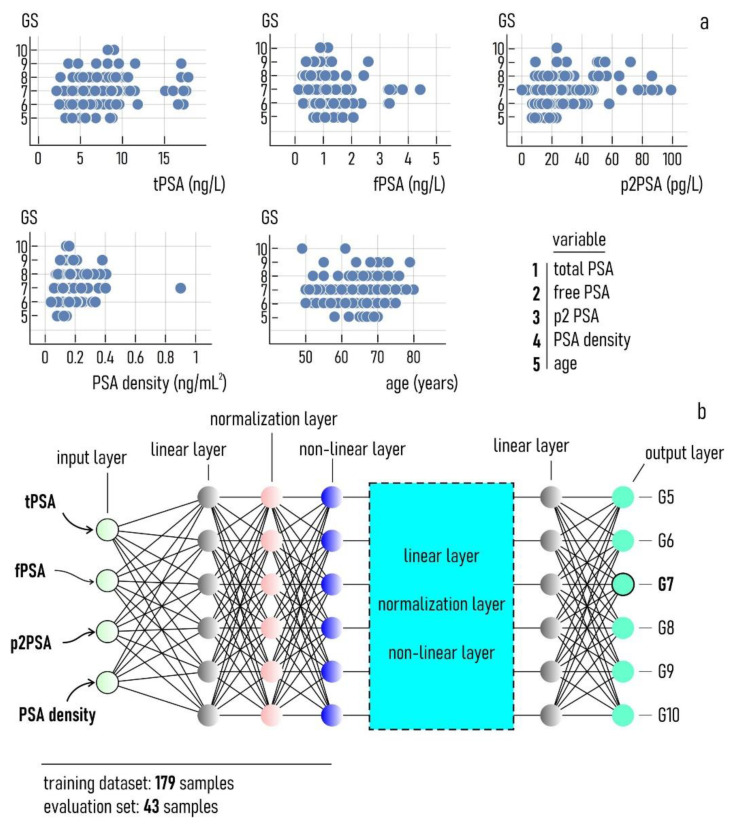
Gleason score reported as a function of several different clinical variables and age used in this study (**a**). The artificial neural network (ANN) used to analyze data and build a predictive model of PC aggressiveness. The network receives as an input the values of the 4 clinical variables of the study. These variables are combined together through successive layers and transmitted to an output layer which determines the grade of the cancer (**b**).

We used a deep-learning algorithm to interpret data and identify aggressive forms of cancer. The algorithm was based on artificial neural networks (ANN) with an input layer, an output layer, and 7 hidden layers in cascade: i.e., a (1) linear layer, (2) normalization layer, (3) hyperbolic tangent non-linear layer, (4) linear layer, (5) normalization layer, (6) hyperbolic tangent non-linear layer, (7) linear layer. Thus, the hidden layers are given by the repetitions of two fundamental units. The input layer was here represented by a set of 4 clinical variables (total PSA, free PSA, precursor of PSA, and PSA density) plus age. The output layer was a vector of 6 integer values ranging from 5 to 10, representing the hypothesized Gleason score of the cancer. In the network, we used 3 neurons for each of the considered variables in each of the layers. The algorithm was implemented in Mathematica. The Mathematica NetInitialize() function was used to instantiate the learning machine. The argument of the function NetInitialize() is the entire sequence of layers that constitute the neural networks model, which are depicted in Figure 2b and here reported for convenience of the reader: NetChain[(Net0, LinearLayer(12), BatchNormalizationLayer(), ElementwiseLayer(Tanh), LinearLayer(6), BatchNormalizationLayer(), ElementwiseLayer(Tanh), LinearLayer(6)]. The command NetChain implies that all the layers are concatenated together. The values between parentheses for each layer represent the number of neurons in that layer, which is identical to that of the immediately preceding layer if left blank. Moreover, LinearLayer, BatchNormalizationLayer, and ElementwiseLayer(Tanh) are the linear, normalization, and non-linear layer, respectively, the latter being based on the hyperbolic tangent function tanh. In this sequence, Net0 is the model input, i.e., the values of concentration of tPSA, fPSA, p2PSA, and PSA density linked together in one layer. The algorithm was trained upon a dataset of 179 samples setting an epoch number of 4000 samples and a batch size of 1024. Thus, the network was trained such that it visited each example 4000 times while processing up to 1024 examples simultaneously. The values of total PSA, free PSA, precursor of PSA, PSA density, and age were concatenated to create a single feature set for each sample. In the tests, the error loss always converged to a value of steady state (10^−2^), indicating that the model achieved optimal performance for the considered values of model parameters. The algorithm was validated against a dataset of 43 samples. We measured the performance of the model using 5 different metrics: (i) accuracy, (ii) precision, (iii) sensitivity, (iv) specificity, and (v) efficiency, where (i) accuracy is the proportion of correct predictions among the total number of examined cases, (ii) precision is the complement to 100 of the average percentage error of the prediction, (iii) sensitivity is the proportion of positive results that are true positives, and (iv) specificity is the proportion of negative results that are true negatives. We define efficiency (v) the mathematical average of accuracy, precision, sensitivity, and specificity.

## 3. Results

### 3.1. The Deep Learning Model

The *GS* against the tPSA, fPSA, p2PSA, PSA density, and age is reported in Figure 2a. These sets of labelled data encode the relationship between the concentration of 4 different biomarkers, plus age, and the Gleason score previously determined through solid biopsy. Remarkably, none of those variables (individually) seem to correlate effectively with the *GS*. For all the cases considered in Figure 2a, values of *r*^2^ statistics far from 1 indicate that data do not match with the predictions of simple linear models. ANNs can find hidden patterns in the data and help to formulate a model that deciphers the relationship between clinical variables and the *GS*. To interpret data, we used the network reported in Figure 2b. The input of the model is a list of values representing the concentration of target biomarkers in blood, and age. The number of variables used as an input was varied to verify their effect on the predictions of the model. The full form of the model uses 5 variables (4 biomarker-derivative variables plus age). The minimum number of variables used in the model was 2, chosen as the combinations of the initial set of 5 variables taking 2 elements at a time. The model achieved maximum performance for an initial set of 4 clinical variables tPSA, fPSA, p2PSA, and PSA density, i.e., the configuration reported in Figure 2b. Adding the variable *age* did not significantly improve the analysis. Results of the model as a function of the number of input variables are shown in the remainder of the paper. In all cases, the output at the last layer is an array of 6 values, from 5 to 10, representing the Gleason score associated with a specific input. The neural network was trained on a database of 190 samples. Then, the model was benchmarked against a validation set of 43 samples.

### 3.2. Best-Case Performance of the Model

**Figure 3:**
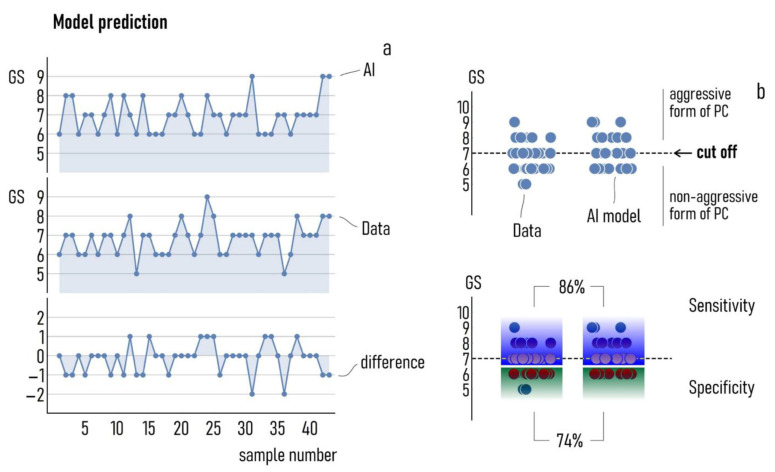
The prediction of the AI model on a cohort of 43 patients used as a validation set compared to the true values of GS measured from those patients (**a**). Using a cut-off value of 7, the model achieved 86% sensitivity and 74% specificity (**b**).

Figure 3a reports the prediction of the model compared to the validation dataset for a basis of 4 input variables (tPSA, fPSA, p2PSA, and PSA density). In 22 out 43 cases, the prediction of the model template matches with the measured *GS*. In 19 cases, the difference between the estimate of the model and the true value of *GS* is of 1 grade. In 2 cases, the *GS* assigned by the model is 2 points higher than the true value of *GS* determined by solid biopsy. Thus, for this configuration the accuracy of the model is ~51%, while the precision is ~93%. Here, we define accuracy as the ratio between the correct predictions and the total number of examined cases, while precision is the complement to 100 of the average percentage errors of the prediction. We then evaluated the performance of the model in identifying aggressive forms of PC. We used = 7 as a cut off value, so that any *GS* ≥ *GS*co indicates the onset of aggressive PC (Figure 3b). We calculated the sensitivity (specificity) of the model test as the proportion of the true positives (negatives) correctly identified. For this choice of input variables, we found that the sensitivity of the model is ~86%, while the specificity is ~74%.

### 3.3. Simulations Predict Patterns of Variables Associated with Aggressive Forms of PC

**Figure 4:**
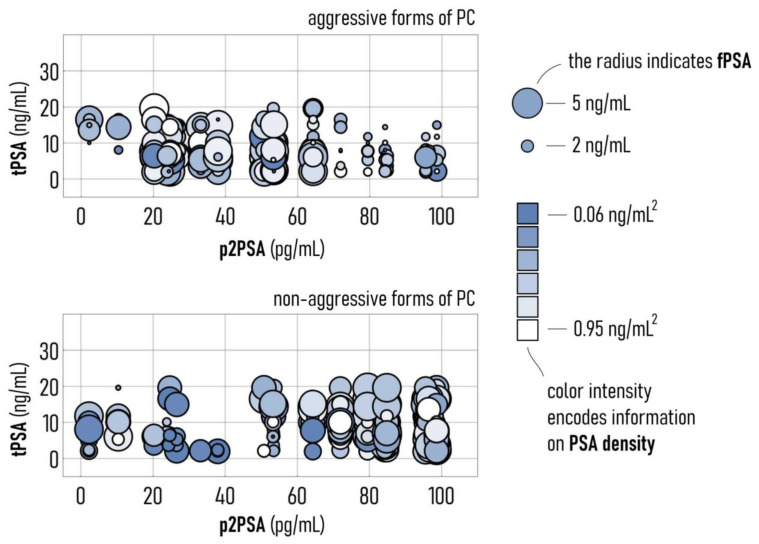
Visual representation of the output of the AI model as a function of the values of the clinical variables tPSA, fPSA, p2PSA, and PSA density. In the upper (lower) row, we report the combination of variables associated with aggressive (non-aggressive) forms of PC, with *GS* ≥ 7 (*GS* < 7).

We then used the model to simulate the more probable *GS* resulting from values of tPSA, fPSA, p2PSA, and PSA density varying over pathological intervals. The values of concentration of tPSA, fPSA, p2PSA, and PSA density were varied between 2.11–17.62 ng/mL (tPSA), 0.1–4.43 ng/mL (fPSA), 0.080–99.12 pg/mL (p2PSA), 0.04–0.9 ng/mL^2^ (PSA density). In this space of variables, we determined those patterns that are associated with aggressive (*GS* ≥ 7) and non-aggressive (*GS* < 7) forms of PC. Results are reported in Figure 4. We represent each combination of 4 variables in the diagrams as disks where the spatial coordinates indicate the concentration of p2PSA and tPSA, the radius of the disk is proportional to the concentration of fPSA, while the color space encodes information about the PSA density. This graphical representation enables us to visualize at a glance several different combinations of clinical variables and the way through which they are correlated to the *GS* of PC. Simulations help to identify 3 domains where the variables have different effects.

In the low p2PSA concentration range (0–20 pg/mL), aggressive forms of PC are correlated to elevated values of tPSA (10–20 ng/mL), sufficiently high values of fPSA (>2 ng/mL), and low values of PSA density (<0.3 ng/mL^2^). For intermediate values of p2PSA concentration (20–70 pg/mL), aggressive forms of PC are correlated to larger values of fPSA (>3 ng/mL) and larger values of PSA density (>0.3 ng/mL^2^), while tPSA seems to have minor effects. For high values of p2PSA concentration (>70 pg/mL), aggressive forms of PC are associated with lower values of tPSA (<10 ng/mL), low values of fPSA (<2 ng/mL), and low values of PSA density (<0.3 ng/mL^2^).

The heterogonous nature of data and the markedly non-linear way in which they interact to yield different values of *GS* reinforce the view that a deep learning approach is necessary to diagnose aggressive forms of PC.

### 3.4. Optimizing Performance: Varying Inputs to the Model

**Figure 5:**
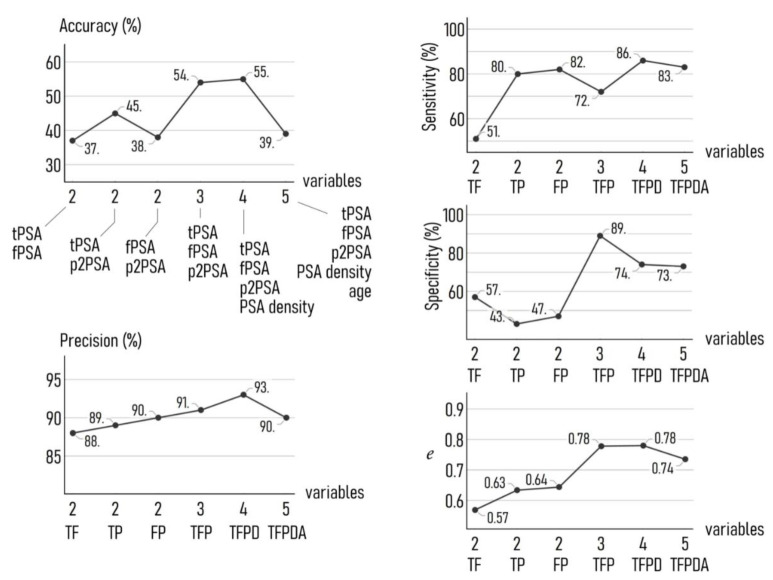
We measured the performance of the model using 4 different metrics, i.e., accuracy (A), sensitivity (Se), specificity (Sp), and precision (P). The error (*e*) is the mean of these 4 metrics. We report in the diagrams the values of A, Se, Sp, P, and *e* determined for the AI model receiving as an input different combinations of the clinical variables used in the study plus age. The model achieved maximum efficiency using as input variables: tPSA, fPSA, p2PSA, and PSA density.

To understand the effects of the input variables we examined how the number and type of variable influences the performance of the model. We developed several different versions of the neuronal networks in which the input variables to the networks were chosen from different combinations of tPSA, fPSA, p2PSA, PSA density, and age. Then, for each combination we evaluated the output of the model in terms of accuracy, precision, sensitivity, specificity, and efficiency, defined as the mathematical average of the 4 last parameters. Results are reported in Figure 5. For notational convenience, in the following we will abbreviate each variable with its initials (tPSA: T, fPSA: F, p2PSA: P, PSA density: D, age: A) so that we will indicate specific combinations of biomarkers with a code.

We observe that accuracy (55%) and precision (93%) are optimized for the set of 4 variables (TFPD). For these estimators, the performance of the model built on an initial set of 3 variables (TFP) is comparable to that measured for 4, with an accuracy of 54% and a precision of 91%. As expected, any combination of 2 variables performs significantly worse than the 3 or 4 variable configuration, with estimated values of accuracy and precision of 45% and 89% for TF, that is the best result among the combinations of T, F, and P. Moreover, the accuracy and precision of the model falls to 39% and 90%, respectively, considering the full set of 5 variables (TFPDA). Considering sensitivity, the combination of 4 variables (TFPD) achieves again the best result, with a value of sensitivity as high as 86%. However, values of sensitivity reported for several other configurations are equivalent to that found for TFPD, with 72% for TFP and 83% for TFPDA. Remarkably, the model scored values of sensitivity equal to 80% and 82% for TP and FP, respectively.

Moving to specificity, we recorded the highest value (89%) for the set of 3 variables TFP, followed by TFPD (74%) and TFPDA (73%). The values of specificity are significantly lower for TF (57%), TP (43%), and FP (47%).

The efficiency (*e*), found as the average of accuracy, precision, sensitivity, and specificity, is a measure of the goodness of the model evaluated all over the other metrics. Values of *e* indicate that the model achieves maximum performance using as input variables TFPD or TFP, for which *e* = 0.78, with a marked increment to the model’s simplest version, that receives as an input 2 sole variables.

## 4. Discussion

Diagnosis and treatment of PC based only on PSA and DRE can lead to overdiagnosis and consequently to overtreatment of indolent cancers [22,23]. Despite the promising results of several new circulating and urinary biomarkers [24] and risk calculators [25], at present no specific biomarker is available that accurately points to biopsy. In order to reduce the side effects of overtreatment, there is a growing need of new diagnostic approach to choose the next step in the clinical management of patients with suspected PC.

Once the presence of malignancy has been established, patients with low-risk cancer have the option of active surveillance (AS) combined with deferred treatment. Such a strategy allows the avoidance of early invasive treatment (radical prostatectomy or radiation therapy) and the associated complications which are harmful for their quality of life. In fact, in 81% of cases patients develop erectile dysfunction, in 17% urinary incontinence, and in 12% bowel dysfunction [26].

The current gold standard to select patients for AS is the use of the D’Amico/NCCN and EAU risk stratification systems or Partin’s table. However, these criteria were developed more than a decade ago when some biomarkers such as −2proPSA were not available. Thus, adding new biomarkers which are better associated with high-grade PC could be an attractive strategy to identify new tools for patient risk stratification.

In the current study we report on the impact of a model including PSA, fPSA, p2PSA, and PSAD on the detection of high-grade PC in an initial biopsy cohort of men aged ≥ 50 years, a representative study population for the impact on the quality of life of possible side effects caused by overtreatment. Total, free, and -2proPSA are used to calculate PHI and there is evidence for PHI and -2proPSA being associated with an increased risk for a positive biopsy and for a Gleason score of ≥7 [27].

In our present study, applying a deep learning strategy, we demonstrated that the addition of PSA density to the three different PSA molecular forms included in PHI calculation showed the highest accuracy and precision for the identification of high-grade PC (*GS* ≥ 7). Our findings confirmed the relevance of PSA molecular forms included in PHI calculation and also highlight the role played by prostate volume in the clinical impact of PC biomarkers. The accuracy and precision obtained for the ANN model including PSA density demonstrated higher values compared to the model when prostate volume was excluded.

Certainly, further investigations are needed to develop new models specifically related to PC aggressiveness to increase current accuracy. However, prostate volume seems to play a key role in the interpretation of laboratory tests for PC [28,29,30]. So, circulating biomarkers related to prostate volume (i.e., PSA and phi density) are worthy of further study. Phi density (phi/prostate volume) outperformed phi in the identification of clinically significant PC in three different single-center studies [31,32,33].

The PSA test is part of routine clinical practice and the measurement of prostate volume is simple; therefore, the application of PSAD to identify high-grade PC would be possible. Accordingly, the recently developed Stockholm-3 model also includes PSA density to obtain the best accuracy to predict clinically significant PC and several recent reports suggest the additional value of PSA density to detect high-risk PC [34,35,36]. Moreover, the addition of PSAD to the criteria used to select patients for active surveillance reduced the rate of biopsy reclassification [37,38].

ANNs have been used to predict prostate cancer outcomes [39,40], but very few models based on circulating biomarkers for predicting the grade of PC are available [41]. Computational intelligence approaches are based on mathematical models able to deal with the uncertainty typically found in the clinical data used for PC prognosis and diagnosis. On this basis, these algorithms represent a suitable platform to develop new strategies for diagnosing and staging PC. For example, not every PC patient will exhibit abnormal results in all biomarkers, so combinations can lead to better diagnostic and prognostic performance [42].

Other authors have explored the potential of this approach. Some researchers [43,44] developed an ANN model for the prognosis of PC, potentially useful to select the patients for biopsy. Shariat et al. [45] suggested that prediction tools can provide help during PC patient clinical decision-making.

Finally, there is evidence that ANN models can be comparable to logistic regression models in predicting PC with clinical significance [46]. Notably, to be available for large populations the panel of reliable biomarkers has to be detected rapidly and at low cost, so development of biosensors [47] together with the use of computational-approach-based models shows relevant potential to face the challenge of selecting patients with clinically significant PC.

There are several limitations to this study. Firstly, it includes data of one population from one area located in Southern Italy, therefore results may not be extrapolated to worldwide population before an external validation. Secondly, our study includes only patients with tPSA between 2 and 20 ng/mL and we did not evaluate how the model performs in patients with tPSA values higher than 20 ng/mL, who are at highest risk of PC. In addition, we consider as outcome GS defined at biopsy, not at radical prostatectomy, thus leaving the identification of high-grade PC under the influence of biopsy sampling error.

However, our present study underlines the promising role of ANN models for the prediction of PC aggressiveness. Currently, the proposed model has been implemented as a research tool, and once prospective validation on a large study population is conducted, the tool will be a simple to use application which can be widely accessible. Such a tool will take the laboratory test results (i.e., PSA, fPSA, p2PSA, and PSAD) of an individual patient and predict his likelihood of having high-grade PC. Therefore, it could aid the clinical decision-making process. In fact, while being more expensive in first-line testing, it could be a tool that may help to reduce costs for the whole clinical management of PC patients, including repeated biopsies, surgery, and drugs for post-surgical complications.

Ongoing work is applying the proposed model to a larger dataset and continuing the search of novel algorithms for predicting aggressive disease. The validation of this model needs to be confirmed on large cohorts prospectively enrolled to assess whether it plays a role in survival, cancer-specific mortality, or metastatic progression.

## 5. Conclusions

In conclusion, in this first reported cohort of men with initial prostate biopsies, an ANN model including PSA, fPSA, p2PSA, and PSA density showed itself to be a good predictor of high-grade biopsy outcome. The investigated model may minimise the risk of missing aggressive cancers, while reducing the number of unnecessary biopsies in men with potentially low-grade disease. The use of our model may have potential in decision making when choosing a treatment strategy that best matches disease aggressiveness and treatment invasiveness.

## Figures and Tables

**Table 1:** Characteristics of the study population.

Patients (*n*)	222
Mean (range, age, years)	64 (50–73)
DRE negative/positive *n* (%)	143/79
Median (95% CI) prostate volume (mL)	50 (45–60)
Gleason score < 7	96/126
tPSA ng/mL	6.35 (4.48–8.38)
fPSA ng/mL	0.89 (0.7–1.3)
p2PSA (pg/mL)	20.29 (14.45–29.52)
PSAD (ng/mL^2^)	0.13 (0.09–0.17)

## Data Availability

The data that support the findings of this study are available on request from the corresponding authors (F.G., D.T.).
